# Predicting miRNA-disease association from heterogeneous information network with GraRep embedding model

**DOI:** 10.1038/s41598-020-63735-9

**Published:** 2020-04-20

**Authors:** Bo-Ya Ji, Zhu-Hong You, Li Cheng, Ji-Ren Zhou, Daniyal Alghazzawi, Li-Ping Li

**Affiliations:** 1 Xinjiang Technical Institutes of Physics and Chemistry, Chinese Academy of Sciences, Urumqi, 830011 China; 2 University of Chinese Academy of Sciences, Beijing, 100049 China; 3 Department of Information Systems, King Abdulaziz University, Jeddah, Saudi Arabia

**Keywords:** Cancer, Computational models

## Abstract

In recent years, accumulating evidences have shown that microRNA (miRNA) plays an important role in the exploration and treatment of diseases, so detection of the associations between miRNA and disease has been drawn more and more attentions. However, traditional experimental methods have the limitations of high cost and time- consuming, a computational method can help us more systematically and effectively predict the potential miRNA-disease associations. In this work, we proposed a novel network embedding-based heterogeneous information integration method to predict miRNA-disease associations. More specifically, a heterogeneous information network is constructed by combining the known associations among lncRNA, drug, protein, disease, and miRNA. After that, the network embedding method Learning Graph Representations with Global Structural Information (GraRep) is employed to learn embeddings of nodes in heterogeneous information network. In this way, the embedding representations of miRNA and disease are integrated with the attribute information of miRNA and disease (e.g. miRNA sequence information and disease semantic similarity) to represent miRNA-disease association pairs. Finally, the Random Forest (RF) classifier is used for predicting potential miRNA-disease associations. Under the 5-fold cross validation, our method obtained 85.11% prediction accuracy with 80.41% sensitivity at the AUC of 91.25%. In addition, in case studies of three major *Human* diseases, 45 (Colon Neoplasms), 42 (Breast Neoplasms) and 44 (Esophageal Neoplasms) of top-50 predicted miRNAs are respectively verified by other miRNA-disease association databases. In conclusion, the experimental results suggest that our method can be a powerful and useful tool for predicting potential miRNA-disease associations.

## Introduction

As a small non-coding RNA (~22nt), MicroRNA (miRNA) plays a lot of critical managerial roles in cells. It is estimated that 1–4% of the genes in the human genome are miRNAs, with individual miRNAs regulating as many as 200 mRNAs^1^. miRNA usually binds to the 3′untranslation regions (UTRs) of the target mRNA through sequence-specific base pairs to inhibit the expression of target mRNA^2–5^. Because of this property, miRNAs can affect various biological processes and participate in a series of important processes in the life process^6–10^. In conclusion, it has been proved that miRNA plays a crucial role in biological processes. Understanding the molecular mechanism of disease is an important goal of biomedical researches. In this post-genome era, more and more contributions made by advanced high-throughput genome technologies are marching toward this goal. A lot of evidence indicates that miRNA plays a vital role in the development and progression of *Human* diseases^1,11–16^. For example, miR-195 expression levels are reduced in patients with Alzheimer’s disease (AD). Besides, over-expression of this miRNA can down-regulate the production of the AD amyloid-β^17^. Moreover, the expression of breast cancer patients’ serum miR-103 levels is significantly higher than that of healthy controls^18^. Therefore, we can believe that miRNA mutations, miRNA biogenic dysfunction, and miRNA’s target disorders may be associated with a variety of diseases, such as lung cancer^19^, lymphoma^20^, breast cancer^21^. However, to our knowledge, compared with a large number of cataloged miRNAs, systematic miRNA-disease association prediction methods are still insufficient. At the same time, the process of traditional laboratory experiments is very expensive and high time-consuming, so it is obvious that the computational method provides a new direction for large-scale miRNA-disease association prediction.

In recent years, a number of computational methods have been proposed to predict the associations between miRNA and disease. These methods can be classified according to their different strategies. For example, You *et al*.^22^ proposed a novel miRNA-disease association prediction model called PBMDA. This model constructs a heterogeneous graph composed of three interrelated subgraphs and then Depth-First-Search (DFS) algorithm is used to predict miRNA-disease associations. Chen *et al*.^23^ proposed a new bipartite network projection model for predicting potential associations between miRNA and disease (BNPMDA) based on miRNA functional similarity, disease semantic similarity, and the known human miRNA-disease associations. Zheng *et al*.^24^ developed a machine learning-based model for miRNA-disease association prediction (MLMDA). This method uses a deep auto-encoder neural network (AE), disease semantic similarity, miRNA sequence information, miRNA functional similarity and Gaussian association spectrum kernel similarity information to predict potential associations between miRNA and disease. Chen *et al*.^25^ established a model called WBSMDA. One of the advantages of this model is that it can be applied to diseases that are not associated with any miRNA, thus breaking through the limitations of most previous methods. You *et al*.^26^ put forward a new calculation method for the prediction of potential associations between miRNA and disease based on a personalized recommendation (PRMDA). In their study, a similarity network was widely used, taking into account the relevant miRNA and disease information for each miRNA-disease pair, thus recommending a high-priority potential miRNA-disease association. Jiang *et al*.^27^ proposed a calculation method to predict potential miRNA-disease associations by prioritizing the human microRNAome for diseases. It is a logical extension of earlier network-based approaches for predicting or prioritizing disease-associated protein-coding genes. They built a functionally-associated miRNA network and a human phenome-microRNAome network to examine whether functionally related miRNAs tended to be associated with diseases with similar phenotypes and prioritize miRNAs for human diseases. Shi *et al*.^28^ proposed a calculation method for miRNA and disease relationship prediction based on random walk analysis. They made a hierarchical clustering analysis on binary miRNA-disease networks to determine the miRNA-disease synergistic control module. Finally, the method yielded a good result, and provided a new perspective for predicting the relationship between miRNA and disease.

**Figure 1:**
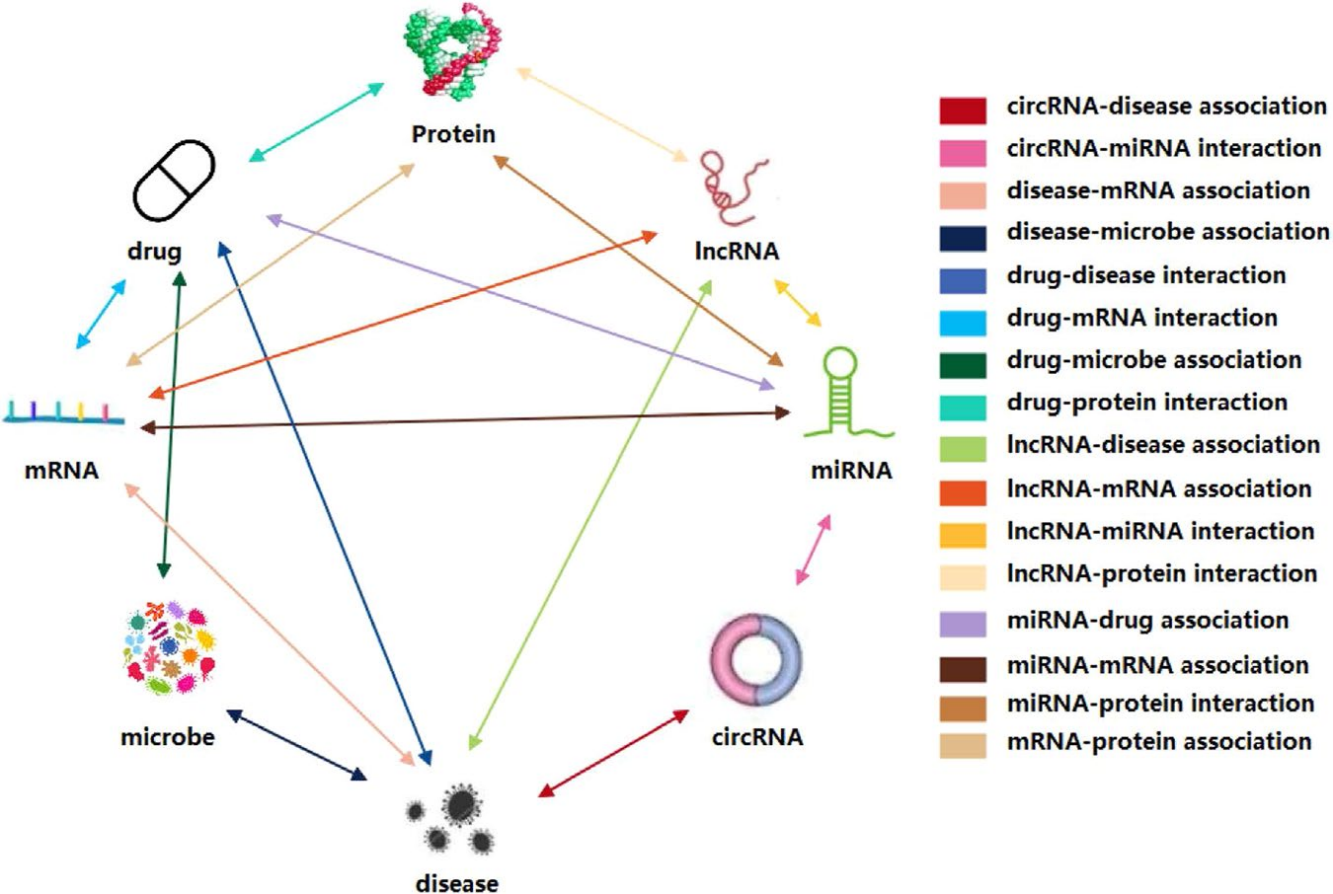
The heterogeneous information network.

**Figure 2:**
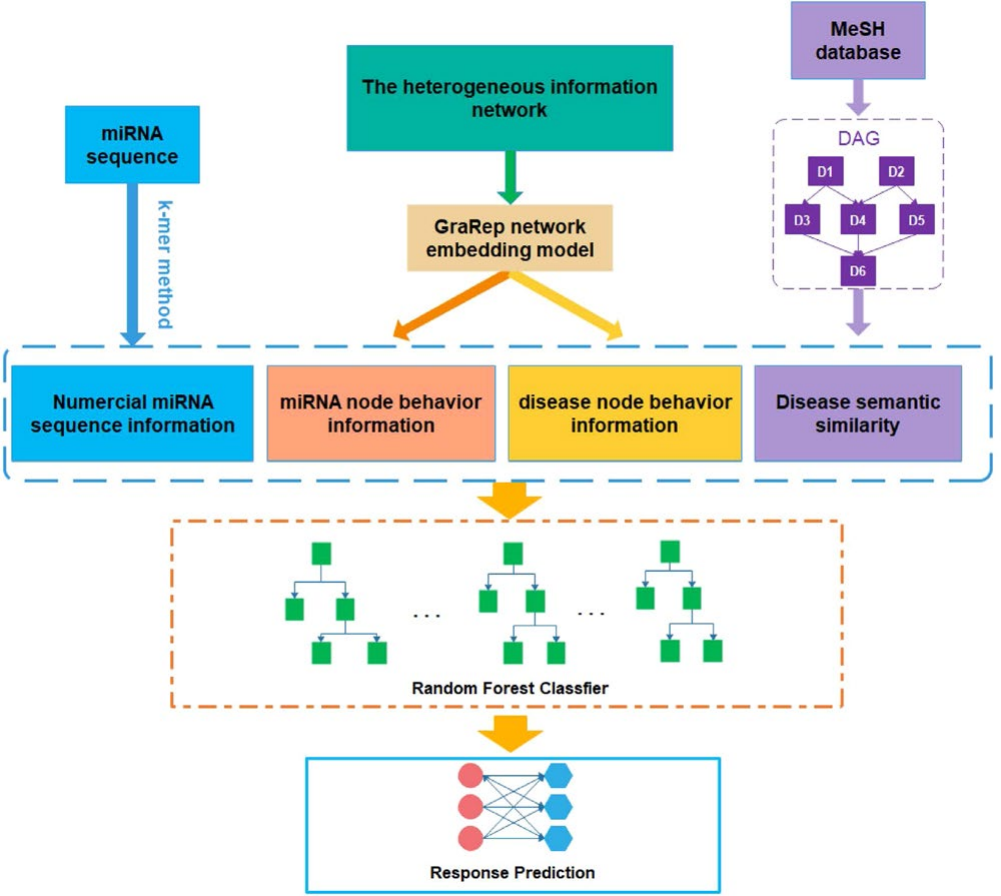
Flowchart of our method to predict potential miRNA-disease associations.

In this study, a network embedding-based heterogeneous information integration method is proposed to predict the potential associations between miRNA and disease. Firstly, a heterogeneous information network is established by combining the known associations between protein, miRNA, lncRNA, disease, and drug as shown in Fig. 1. After that, the network embedding method GraRep is adopted to learn the behavior information of miRNA and disease node in the network. As one of the network representation learning (NRL) models, the GraRep method can learn graph representations of the miRNA and disease nodes with global structural information. Secondly, the miRNA and disease nodes were converted to a vector by integrating the attribute information of the node itself (miRNA sequence information and disease semantic similarity) and the behavior information of them in the network to represent miRNA-disease pairs. Thirdly, 16427 known miRNA and disease pairs, which obtained from HMDD^29^ database, are used as positive samples and the same number of unrelated miRNA and disease pairs are randomly selected as negative samples, the two kinds of samples are combined to form the training samples. Finally, the prediction models are constructed based on the training samples by using the random forest, Fig. 2 shows the flowchart of our method. The model was evaluated through the 5-fold cross validation, and it performs well with high accuracy. To further test the effect of our method, we also conducted case studies of three major *Human* diseases. Our experiments prove that the network embedding method has great potential and provides a new direction for the prediction of miRNA and disease associations.

## Materials and Methods

### Heterogeneous information network construction

To systematically and comprehensively build the network of heterogeneous information, some known associations between miRNAs, lncRNAs, proteins, diseases, and drugs from multiple databases were downloaded. The source and version of the raw data are shown below: The miRNA-lncRNA association pairs are downloaded from the lncRNASNP2 database of Miao *et al*.^30^. The miRNA-protein association pairs are downloaded from the miRTarBase update 2018 database of Chou *et al*.^31^. The lncRNA-disease association pairs are downloaded from the lncRNASNP2 and LncRNADisease database of Miao *et al*.^30^ and Chen *et al*.^32^. The drug-disease association pairs are downloaded from the comparative toxicogenomics database: update 2019 of Davis *et al*.^33^. The lncRNA-protein association pairs are downloaded from the LncRNA2Target v2.0 database of Cheng *et al*.^34^. The drug-protein association pairs are downloaded from the DrugBank 5.0 database of Wishart *et al*.^35^. The protein-protein association pairs are downloaded from the STRING database in 2017 of Szklarczyk *et al*.^36^. The protein-disease association pairs are downloaded from the DisGeNET database of Piñero *et al*.^37^. The miRNA-disease association pairs are downloaded from the HMDD v3.0 database of Huang *et al*.^29^. After that, a series of operations such as unifying identifiers, de-redundancy, simplifying and deleting irrelevant items are conducted. The detailed data of the final experiment is shown in Table 1. In addition, we also classify and sort the above associations. Finally, we get different nodes as shown in Table 2.

**Table 1:** The associations in the heterogeneous information network.

Association type	Database	Number of associations
miRNA-lncRNA	lncRNASNP2^30^	8374
miRNA-protein	miRTarBase: updata 2018^31^	4944
lncRNA-disease	LncRNADisease^32^,	
	lncRNASNP2^30^	1264
drug-disease	CTD: updata 2019^33^	18416
lncRNA-protein	LncRNA2Target v2.0^34^	690
drug-protein	DrugBank v5.0^35^	11107
protein-protein	STRING: in 2017^36^	19237
protein-disease	DisGeNET^37^	25087
Total	N/A	105546

**Table 2:** The nodes in the heterogeneous information network.

Node	Amount
Protein	1649
Disease	2062
LncRNA	769
Drug	1025
MiRNA	1023
Total	6528

### Numerical miRNA sequence information

The sequences of miRNA are downloaded from miRbase^38^, to represent the attribute information of the miRNA node. To make the experiment less complicated, we select the 3-mer method and encode the miRNA sequence into a 64-dimensional feature vector, where each component represents the frequency of the occurrence of a 3-mer in the sequence (e.g. UGC, AUC, GUA).

### Disease semantic similarity

**Figure 3:**
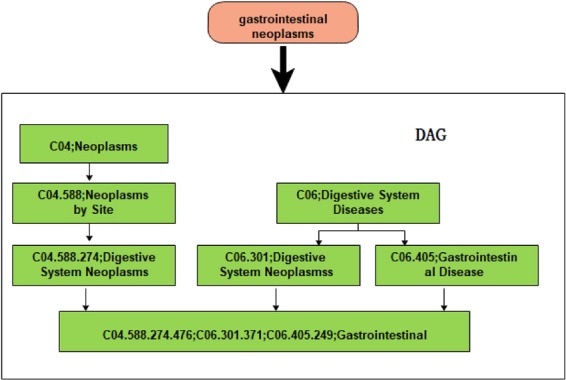
Construction of gastrointestinal neoplasms’ DAG.

The Medical Subject Heading (MeSH) database is a strict disease classification system, which can be used to effectively study the relationship between different diseases. Through this system, we can represent each disease with the Directed Acyclic Graph (DAG) achieved by MeSH of it. For example, for disease A, we can represent it as DAG(A) = (A, T(A), E(A)), where T(A) denotes all nodes in the DAG(A) that contain the disease A, E(A) indicating all disease link relationships in DAG(A)^39^. An example of gastrointestinal neoplasms’ DAG is shown in Fig. 3 below:

Therefore, we can select the disease semantic similarity calculated by DAG as the attribute information of disease according to the earlier method^39^. The semantic value of a disease D can be calculated as follows:

1 $${\rm{DV}}({\rm{D}})=\sum _{d\in T(D)}{D}_{D}(d)$$

2 $$\{\begin{array}{l}{D}_{D}(d)=1\,if\,d=D\\ {D}_{D}(d)=max\{{\Delta }_{\ast }{D}_{D}({d}^{\text{'}})|{d}^{\text{'}}\in {\rm{children}}\,{\rm{of}}\,{\rm{d}}\}\,if\,d\ne D\end{array}$$

where ∆ is the semantic contribution factor and T(D) represents D and its all ancestor nodes. Observed results show that the two similar DAG ratios have higher disease similarity and the semantic similarity for disease ${d}_{i}$ and ${d}_{j}$ are defined as follows:

3 $${\rm{SS}}({d}_{i},{d}_{j})=\frac{{\sum }_{t\in T({d}_{i}){\cap }^{}T({d}_{j})}(D{1}_{{d}_{i}}(t)+D{1}_{{d}_{j}}(t))}{DV1({d}_{i})+DV1({d}_{j})}$$

### Stacked autoencoder

For the purpose of reducing the noise in the attribute information and normalizing it in a uniform dimension, we use a stacked autoencoder (SAE) to transform the original feature space into an appropriate subspace. SAE mainly consists of the following two steps: 1, the encoder projects *x* from the input layer to the hidden layer *h* through a mapping function *f*. 2, The decoder maps *h* in the hidden layer to *y* in the output layer through a mapping function *g*.

4 $${\rm{h}}={\rm{f}}({\rm{x}}):={S}_{f}(Wx+p)$$

5 $${\rm{y}}={\rm{g}}({\rm{h}}):={S}_{g}(W{\prime} x+q)$$

In this study, the ReLU function was selected as the activation function:

6 $${S}_{f}(t)={S}_{g}(t)=\,{\rm{\max }}(0,Wt+b)$$

### GraRep algorithms

Recently, many Network Representation Learning (NRL) methods have been proposed to learn vector representations of vertices in a network. GraRep^40^ is one of these methods. It factorizes different *k*-order proximity matrices and concatenates the embeddings learned from each proximity matrix. Specifically, GraRep takes into consideration the special relation matrix and extends the skip-gram model to capture the high order proximity of a network. It defines the *k*-step neighbors (*k* ≥ 1), and nodes that share a common *k*-step neighbor in the network should have similar and potential representations. Formally, the *k*-step representation of the learning node is composed of three steps. The first step is to obtain the *k*-step transition probability matrix ${A}^{k}$ for each *k* = 1, 2, … K. The second step is to use SVD method to factor the logarithmic probability matrix ${X}^{k}$ to obtain each *k* step representation:

7 $${X}^{k}={U}^{k}{{\sum }^{}}^{k}{({{\rm{V}}}^{{\rm{k}}})}^{T}$$

where both *U* and *V* are orthonormal matrices and *∑* is a diagonal matrix that consists of an ordered list of singular values. The third step is to connect all *k* step representations, which can be represented as the following matrix:

8 $$W=[{W}^{1},{W}^{2},{W}^{3},\ldots {W}^{k}]$$

More detailed algorithmic process participation can be seen in Table 3.

**Table 3:**
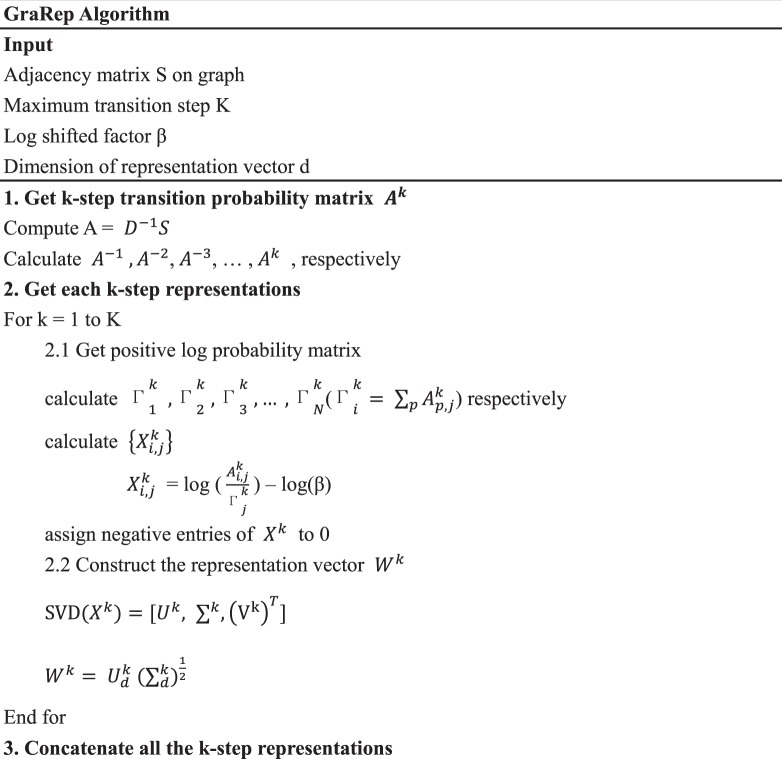
The GraRep Overall Algorithm.

### Node representation

The miRNA and disease nodes are represented by their intrinsic attribute information and behavior information with other nodes in the heterogeneous information network. The attribute information is respectively numerical miRNA sequence information and disease semantic similarity. In addition, in this paper, a network embedding method GraRep is used to obtain the behavior information of nodes in the entire network, before combining with their own attribute information. Their relationship with other nodes can be regarded as a functional representation based on the idea of collaborative filtering. Finally, they are converted into 128-dimensional vectors to represent known miRNA-disease associations.

## Result and Discussion

### Evaluate the performance of our method under the 5-fold cross validation

5-fold cross validation was used to evaluate the performance of our study, which randomly divided all data sets into five equal parts. In each validation, one part is used as the test set and the other four parts as the training set, so that test and training data do not overlap each other to ensure unbiased comparisons. The detailed result information of the proposed method is shown in Table 4. It can be seen from Table 4 that our proposed method exhibited the outcomes of average accuracy (Acc.), precision (Prec.), sensitivity (Sen.), matthews correlation coefficient (MCC), specificity (Spec.) and the areas under the ROC curve (AUC) of 85.11%, 88.75%, 80.41%, 70.53%, 89.81% and 91.25%, respectively.

**Table 4:** The performance of our method under 5-fold cross validation.

Fold	ACC.(%)	Prec.(%)	Sen.(%)	MCC(%)	Spec.(%)	AUC(%)
0	85.29	89.00	80.52	70.89	90.05	91.32
1	85.23	89.17	80.19	70.81	90.26	91.24
2	84.57	88.51	79.46	69.51	89.68	90.66
3	85.54	88.68	81.50	71.32	89.59	91.51
4	84.92	88.41	80.38	70.13	89.46	91.53
**Average**	**85.11 ± 0.37**	**88.75 ± 0.32**	**80.41 ± 0.73**	**70.53 ± 0.71**	**89.81 ± 0.33**	**91.25 ± 0.35**

**Figure 4:**
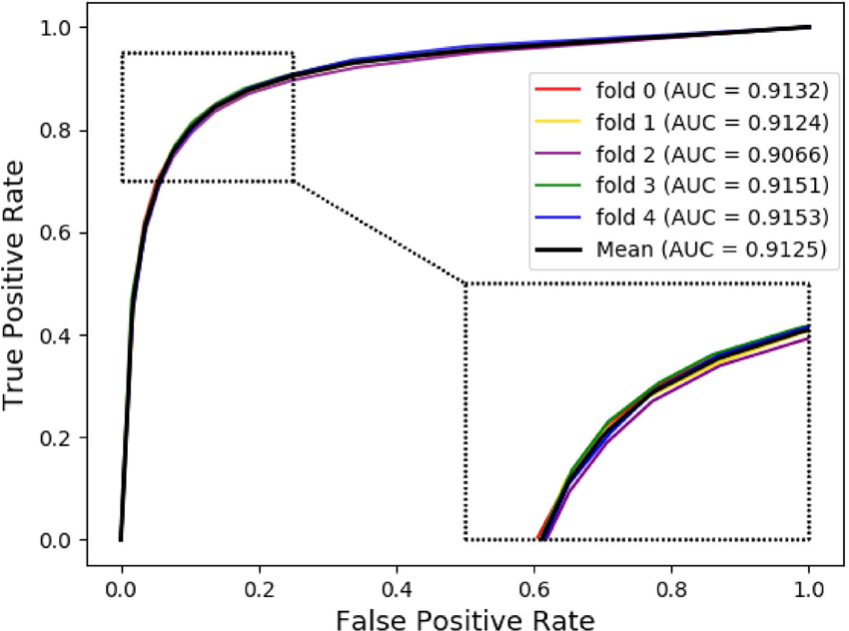
The ROC curves of our method in miRNA-disease association prediction under 5-fold cross validation.

**Figure 5:**
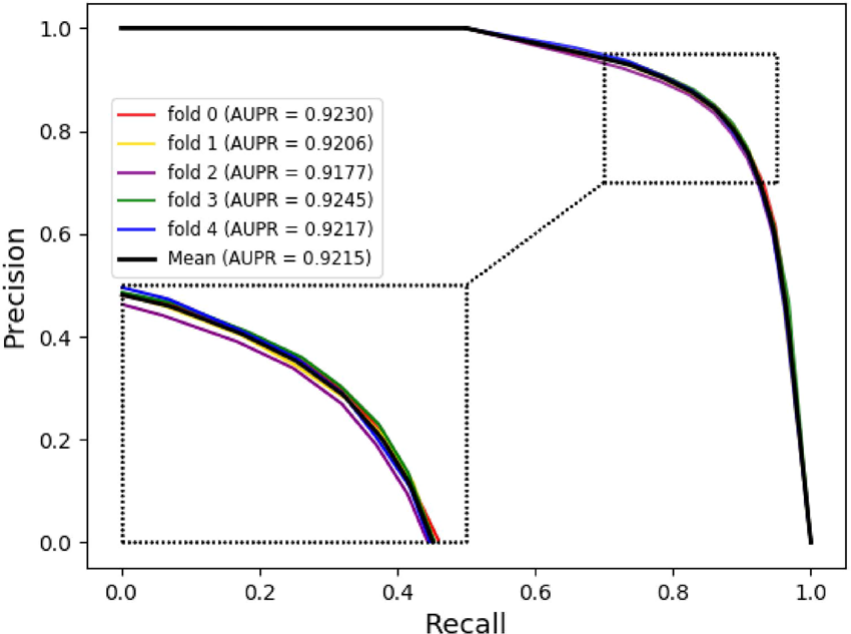
The PR curves of our method in miRNA-disease association prediction under 5-fold cross validation.

The receiver operating characteristic (ROC) curve is a functional image describing sensitivity. Here, its horizontal axis represents the False Positive Rate (FPR), which represents the ratio of all negative examples in the partitioning example to all negative cases (1-Specificity), where the larger the FPR and the more positive negative classes in the positive class are predicted. Besides, its vertical axis represents the True Positive Rate (TPR), which is used to represent the positive class coverage (Sensitivity). The larger the TPR and the more positive classes in the positive class are predicted. The AUC value indicates the areas under the ROC curve and it ranges from 0.1 to 1. AUC can be used as a numerical value to directly evaluate the quality of the classifier. We can see from Fig. 4 that the average AUC value obtained by our method is 0.9125. The Precision-Recall (PR) curve is another way to evaluate the performance of our method. It shows a trade-off between precision and sensitivity for all possible thresholds. From Fig. 5, we can see the PR curve corresponding to our method and the mean of the area under the precision-recall curve (AUPR) value is 0.9215. This once again proves that the good performance of our method.

### Comparison of our method with different feature combinations

**Figure 6:**
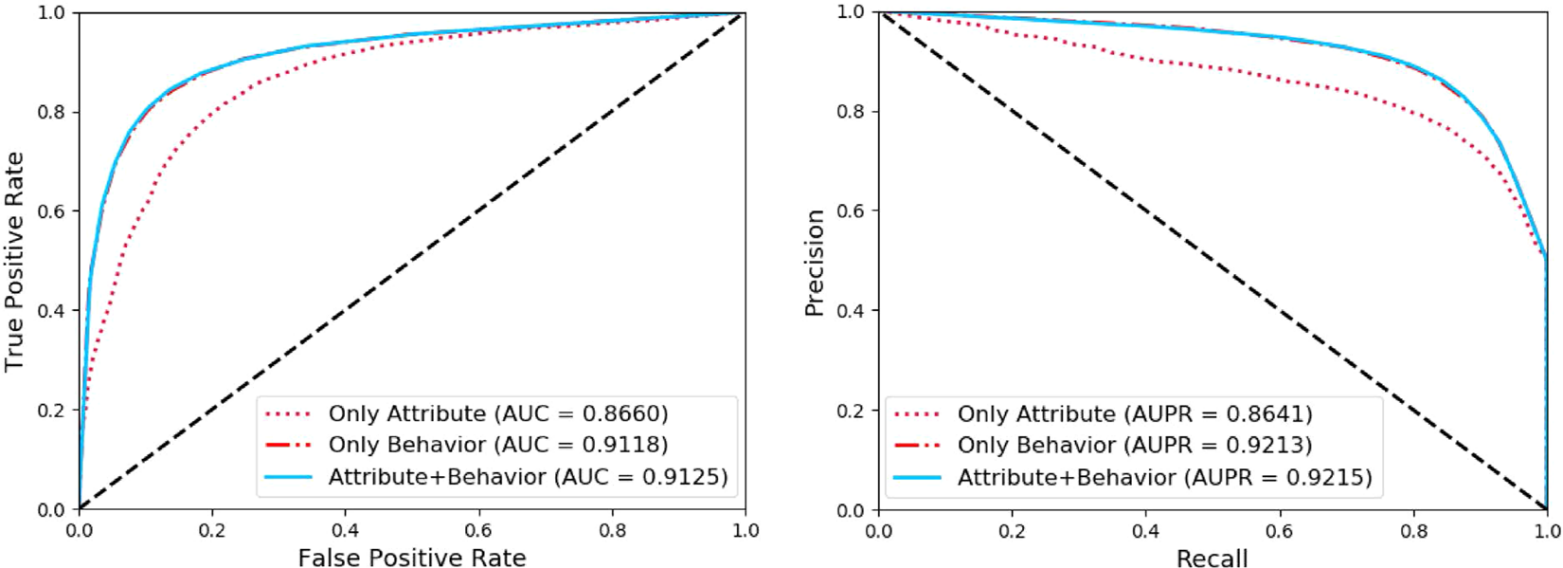
Comparison of our method with different features under 5-fold cross validation.

As we stated above, we use two different pieces of information to represent miRNA and disease in the entire network. Therefore, for the purpose of further testing the influence of various types of feature combinations on the classification results, we use attribute information, behavior information and attribute information plus behavior information to represent nodes respectively before conducting 5-fold cross-validation experiments. As the results of the final experiment shown in Table 5 and Fig. 6, there is a better performance in classification when we consider the attribute and behavior information simultaneously.

**Table 5:** Comparison of our method with different feature combinations.

Feature	Acc.(%)	Prec.(%)	Sen.(%)	MCC(%)	Spec.(%)	AUC(%)
Attribute	79.77 ± 0.42	78.77 ± 0.61	81.52 ± 0.47	59.59 ± 0.83	78.03 ± 0.82	86.60 ± 0.37
Behavior	85.00 ± 0.28	88.42 ± 0.29	80.54 ± 0.86	70.27 ± 0.50	89.45 ± 0.39	91.18 ± 0.32
**Both**	**85.11 ± 0.37**	**88.75 ± 0.32**	**80.41 ± 0.73**	**70.53 ± 0.71**	**89.81 ± 0.33**	**91.25 ± 0.35**

### Comparison of our method with different classifiers

**Figure 7:**
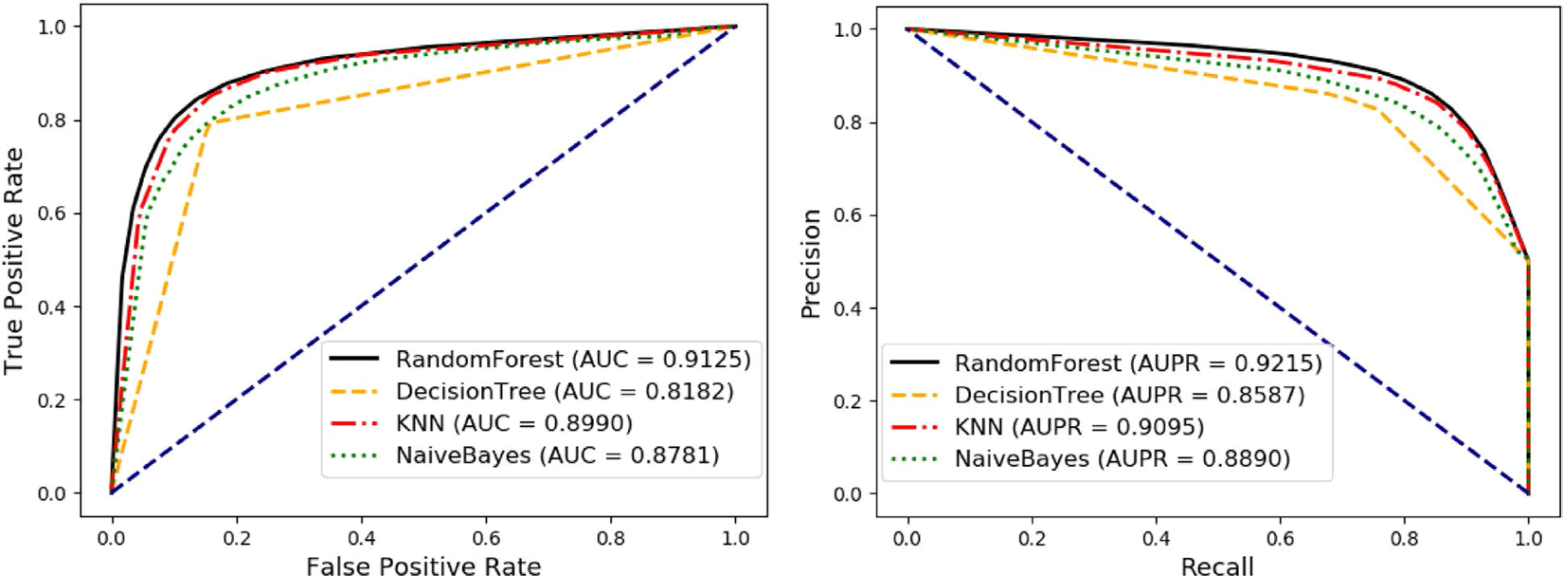
Comparison with Random Forest, DecisionTree, KNN, and Naive Bayes classifier under 5-fold cross validation.

To further test the influence of the classifier in our model, we compared the performance of the four classifiers of Random Forest^41^, Decision Tree^42^, KNN^43^, and Naive Bayes^44^ under 5-fold cross validation. During the comparison experiment, we kept the same experimental environment, same training set and test set, and only changed the type of classifier. Similarly, we still use the six parameters: accuracy (Acc.), precision (Prec.), sensitivity (Sen.), matthews correlation coefficient (MCC), specificity (Spec.), and the areas under the ROC curve (AUC) as evaluation indicators. In the result, the Random Forest model yields average Acc., Prec., Sen., MCC, Spec. and AUC of 85.11 ± 0.37%, 88.75 ± 0.32%, 80.41 ± 0.73%, 70.53 ± 0.71%, 89.81 ± 0.33% and 91.25 ± 0.35%. Table 6 and Fig. 7 show the final comparison results. It can be seen that the Random Forest classifier has better performance and robustness than other classifiers, especially in the accuracy and AUC that can more represent the performance of the model, although our model is not as good as KNN and Naïve Bayes model are in sensitivy. In short, Random Forest is a better classifier for our model.

**Table 6:** Comparison of our method with different classifiers.

Classifier	ACC.(%)	Prec.(%)	Sen.(%)	MCC.(%)	Spec.(%)	AUC.(%)
DecisionTree	81.82 ± 0.23	83.59 ± 0.41	79.18 ± 0.11	63.72 ± 0.47	84.45 ± 0.47	81.82 ± 0.23
KNN	84.62 ± 0.47	84.23 ± 0.37	85.18 ± 0.86	69.24 ± 0.94	84.06 ± 0.42	89.90 ± 0.39
Naive Bayes	81.79 ± 0.67	81.02 ± 0.85	83.04 ± 0.51	63.61 ± 1.34	80.54 ± 1.01	87.81 ± 0.55
**RandomForest**	**85.11 ± 0.37**	**88.75 ± 0.32**	**80.41 ± 0.73**	**70.53 ± 0.71**	**89.81 ± 0.33**	**91.25 ± 0.35**

### Case studies

In order to further test the prediction accuracy of our method, three *Human* diseases are selected for case studies. They are Colon neoplasms, Breast neoplasms, and Esophageal neoplasms, which are closely related to human health. We utilized the known miRNA-disease associations in HMDD V3.0^29^ as the training set. The embedding representations of miRNA and disease are integrated with the attribute information of them (e.g. miRNA sequence information and disease semantic similarity) to represent these known miRNA-disease association pairs so that the input miRNAs and diseases can be identified by the classifier. Finally, the prediction model is constructed based on the training set by using random forest. After that, we constructed the test set for each investigated disease. The test set contains miRNAs in the heterogeneous information network and corresponding disease association pairs. In particular, the miRNA-disease association pairs already existing in the training set were deleted in the test set, including the disease-related miRNAs listed in Tables 7–9. Similarly, after converting the test set into the combination of embedding representations and attribute information, we use the prediction model to make predictions. After the completion of the prediction, the top 50 predicted miRNAs are selected and validated using two other miRNA-disease association databases, dbDEMC^45^ and miR2Disease^46^.

**Table 7:** Predicted the top 50 miRNAs associated with colon neoplasms. The first column recorded the top 1–25 associated miRNAs. The second column recorded the top 26–50 associated miRNAs.

miRNA	Evidence	miRNA	Evidence
hsa-mir-186-5p	dbDemc	hsa-mir-129-5p	dbDemc
hsa-mir-16-5p	dbDemc	hsa-mir-503-5p	dbDemc
hsa-mir-485-5p	dbDemc	hsa-mir-136-5p	dbDemc
hsa-mir-497-5p	dbDemc	hsa-mir-324-5p	dbDemc
hsa-mir-206	dbDemc;miR2Disease	hsa-mir-10a-5p	dbDemc
hsa-mir-33b-5p	dbDemc	hsa-mir-199a-5p	dbDemc
hsa-mir-19b-3p	dbDemc	hsa-mir-199b-5p	dbDemc
hsa-mir-198	dbDemc;miR2Disease	hsa-mir-451a	dbDemc
hsa-mir-361-5p	dbDemc	hsa-mir-29c-5p	dbDemc
hsa-mir-185-5p	dbDemc	hsa-mir-181a-2-3p	dbDemc
hsa-mir-154-5p	dbDemc	hsa-mir-184	dbDemc;miR2Disease
hsa-mir-26b-5p	dbDemc	hsa-mir-99b-5p	dbDemc
hsa-mir-638	dbDemc;miR2Disease	hsa-mir-144-5p	dbDemc
hsa-mir-34c-5p	dbDemc	hsa-mir-128-1-5p	dbDemc
hsa-mir-122-5p	dbDemc	hsa-mir-92a-2-5p	dbDemc
hsa-mir-449b-5p	dbDemc	hsa-mir-337-5p	dbDemc
hsa-mir-590-5p	dbDemc	hsa-mir-423-5p	dbDemc
hsa-mir-139-5p	dbDemc	hsa-mir-663a	dbDemc
hsa-mir-340-5p	dbDemc	hsa-mir-99a-5p	Unconfirmed
hsa-mir-542-5p	dbDemc;miR2Disease	hsa-mir-378a-5p	dbDemc
hsa-mir-211-5p	dbDemc	hsa-mir-575	dbDemc
hsa-mir-153-3p	Unconfirmed	hsa-mir-373-5p	Unconfirmed
hsa-mir-149-5p	dbDemc	hsa-mir-214-5p	dbDemc
hsa-mir-499a-5p	Unconfirmed	hsa-mir-217-5p	Unconfirmed
hsa-mir-183-5p	dbDemc	hsa-mir-452-5p	dbDemc

**Table 8:** Predicted the top 50 miRNAs associated with esophageal neoplasms. The first column recorded the top 1–25 associated miRNAs. The second column recorded the top 26–50 associated miRNAs.

miRNA	Evidence	miRNA	Evidence
hsa-mir-182-5p	dbDemc	hsa-mir-181d-5p	dbDemc
hsa-mir-186-5p	dbDemc	hsa-mir-449a	dbDemc
hsa-mir-30e-5p	dbDemc	hsa-mir-140-5p	dbDemc
hsa-mir-107	dbDemc	hsa-mir-590-5p	dbDemc
hsa-mir-16-5p	dbDemc	hsa-mir-29b-3p	dbDemc
hsa-mir-195-5p	dbDemc	hsa-mir-134-5p	dbDemc
hsa-mir-103a-3p	dbDemc	hsa-mir-24-3p	dbDemc
hsa-mir-15b-5p	dbDemc	hsa-let-7e-5p	dbDemc
hsa-mir-206	dbDemc	hsa-mir-125a-5p	dbDemc
hsa-mir-30a-5p	dbDemc	hsa-mir-153-3p	dbDemc
hsa-mir-18a-5p	dbDemc	hsa-mir-149-5p	dbDemc
hsa-mir-135a-5p	dbDemc	hsa-mir-221-5p	Unconfirmed
hsa-mir-33a-5p	dbDemc	hsa-mir-152-5p	Unconfirmed
hsa-mir-17-5p	dbDemc	hsa-mir-204-5p	dbDemc
hsa-mir-19b-3p	dbDemc	hsa-let-7f-5p	dbDemc
hsa-mir-20b-5p	dbDemc	hsa-let-7d-5p	dbDemc
hsa-mir-106a-5p	dbDemc	hsa-mir-504-5p	dbDemc
hsa-mir-7-5p	dbDemc	hsa-mir-129-5p	dbDemc
hsa-mir-26a-5p	dbDemc	hsa-mir-144-5p	Unconfirmed
hsa-mir-9-5p	dbDemc	hsa-mir-324-5p	dbDemc
hsa-mir-181b-5p	dbDemc	hsa-mir-191-5p	dbDemc
hsa-mir-181a-5p	dbDemc	hsa-mir-199a-5p	dbDemc
hsa-mir-1271-5	Unconfirmed	hsa-mir-29a-5p	Unconfirmed
hsa-mir-122-5p	dbDemc	hsa-mir-125b-2-3p	dbDemc
hsa-mir-181c-5p	dbDemc	hsa-mir-127-5p	Unconfirmed

**Table 9:** Predicted the top 50 miRNAs associated with breast neoplasms. The first column recorded the top 1–25 associated miRNAs. The second column recorded the top 26–50 associated miRNAs.

miRNA	Evidence	miRNA	Evidence
hsa-mir-186-5p	dbDemc	hsa-mir-508-5p	dbDemc
hsa-mir-539-5p	dbDemc	hsa-mir-525-5p	Unconfirmed
hsa-mir-216a-5p	dbDemc	hsa-mir-431-5p	dbDemc
hsa-mir-330-5p	dbDemc	hsa-mir-532-5p	dbDemc
hsa-mir-154-5p	dbDemc	hsa-mir-483-5p	dbDemc
hsa-mir-543	dbDemc	hsa-mir-519a-5p	Unconfirmed
hsa-mir-181d-5p	dbDemc	hsa-mir-581	dbDemc
hsa-mir-4262	Unconfirmed	hsa-mir-744-5p	dbDemc
hsa-mir-449b-5p	dbDemc	hsa-mir-362-5p	dbDemc
hsa-mir-384	dbDemc	hsa-mir-432-5p	dbDemc
hsa-mir-211-5p	dbDemc	hsa-mir-511-5p	dbDemc
hsa-mir-4458	dbDemc	hsa-mir-513b-5p	dbDemc
hsa-mir-504-5p	dbDemc	hsa-mir-513c-5p	dbDemc
hsa-mir-28-5p	dbDemc	hsa-mir-583	dbDemc
hsa-mir-1271-5p	dbDemc	hsa-mir-628-5p	dbDemc
hsa-mir-136-5p	dbDemc	hsa-mir-939-5p	dbDemc
hsa-mir-300	dbDemc	hsa-mir-885-5p	Unconfirmed
hsa-mir-99b-5p	dbDemc	hsa-mir-1973	Unconfirmed
hsa-mir-337-5p	dbDemc	hsa-mir-369-5p	dbDemc
hsa-mir-518b	Unconfirmed	hsa-mir-612	Unconfirmed
hsa-mir-637	dbDemc;miR2Disease	hsa-mir-665	dbDemc
hsa-mir-217-5p	Unconfirmed	hsa-mir-943	dbDemc
hsa-mir-517a-3p	dbDemc	hsa-mir-490-5p	dbDemc
hsa-mir-646	dbDemc	hsa-mir-188-5p	dbDemc
hsa-mir-671-5p	dbDemc	hsa-mir-942-5p	dbDemc

Colon neoplasms is a common malignant tumor in the gastrointestinal tract. As the most common part of colorectal cancer, it has an incidence rate which is second only to gastric and esophageal cancer. At the same time, as one of the most famous tumors, it plays a vital role in gene and cell growth. Moreover, since the early performance of colon neoplasms is not obvious, many patients have reached the late stage of its discovery so that they missed the best treatment opportunity^47^. More seriously, more and more studies have shown that patients with colon neoplasms disease are on the increase year by year^48^. In addition, the associations between miRNA and colon neoplasms has been discovered and confirmed by more and more experimental researchers, which proves once again that miRNA plays an important role in colon neoplasms. Therefore, there is an urgent need to predict the potential miRNA associated with colon neoplasms. For example, miR-143 and miR-145 are both confirmed to continue to be downregulated during colon neoplasms production^12^. In addition, miR-17 and miR-106a, which have been deleted in colon neoplasms and shown to use E2F1 as a target mRNA and inhibit the growth of colon neoplasms^49^. Therefore, we selected colon neoplasms as a case study to further test the accuracy of our method for the purpose of predicting potential miRNA-disease associations. According to dbDEMC and miR2Disease’s evidence, 45 of the top 50 predicted miRNAs are successfully confirmed (see Table 7). For example, the association between hsa-miR-206 and colon neoplasms has been confirmed by previous literature^50^. This method found that hsa-miR-206 can participate in the targeting and regulation of SLC44A1 and KLF13, thus participate in the occurrence and metastasis of colon cancer.

Esophageal neoplasms is another epidemic cancer, which is a deadly disease and one of the most common digestive tract tumors^51^. Its prevalence is due to the current poor eating habits. At present, research on it is still rare in the world. The most common symptom of patients with esophageal neoplasms is dysphagia, which can lead to pain, vomiting, weight loss, etc^52^. The most common method currently used for this disease is chemotherapy. Where appropriate, chemotherapy allows patients to achieve the longest remission period and prolong the survival of some patients. Some studies have shown that miRNAs can be considered as effective prognostic biomarkers for esophageal neoplasms^53^. Therefore, case studies of Esophageal Neoplasms were conducted on our method to select the most likely-associated miRNAs. According to dbDEMC and miR2Disease’s evidence, 44 of the top 50 predicted miRNAs were verified (see Table 8). For example, the association between hsa-miR-182-5p and esophageal neoplasms has been confirmed by previous method^54^. This method identified two new tumor suppressor miRNA, including miR-182-5p and miR-455-5p, of which has-miR-182-5p was confirmed to be associated with esophageal cancer.

Breast neoplasms is a kind of malignant tumor formed by the uncontrolled growth of abnormal breast cells^55^. Each year, more than 211,000 cases of invasive breast cancer are diagnosed in the United States^56^. In most cases, breast cancer occurs in women, but it can also occur in men. More than 1,600 cases of male breast cancer are diagnosed each year. Breast cancer in women remains a major medical problem with major public health and social implications. At present, breast cancer has posed a threat to women’s physical and mental health^57^. In addition, numerous experiments have proved that many miRNAs are related to breast neoplasms. Case studies of Breast Neoplasms were conducted on our method to select the most likely-associated miRNAs. According to dbDEMC and miR2Disease’s evidence, 42 of the top 50 predicted miRNAs were verified (see Table 9).

## Conclusions

Prediction of the associations between miRNA and disease can not only help us better understand the important role of miRNA in the generation and development of diseases, but also greatly promote the diagnosis and treatment of diseases. In this article, we proposed a new method to predict the potential associations between miRNA and disease by extracting the embedding representation of miRNAs and diseases from the heterogeneous information network. After that, we used the GraRep method to get the behavior information of miRNAs and disease in the network before combining their attribute information to represent miRNA and disease nodes, respectively. Then, we put the final data set into the Random Forest classifier for training and prediction. The final experimental results show that our method performs well and it is better than the methods of using only attribute information and methods using only behavior information. In addition, the results of the case study also prove that our method can predict the potential miRNA-disease associations well and the associated miRNA of a given disease. Therefore, we believe that the proposed method will be a useful and efficient tool for predicting miRNA-disease associations in the future. Besides, the working code explored in this article is available at https://github.com/jiboya123/working-code.git.
